# The Treatment of Uterine Tumours by Electricity

**Published:** 1890-03

**Authors:** 


*The Treatment of Uterine Tumours by Electricity.* By THOMAS KEITH, M.D., LL.D., and SKENE KEITH, F.R.C.S. Pp. 255. Edinburgh: OLIVER & BOYD. 1889.

The title of this work is perhaps a little misleading. The author is concerned, not with uterine tumours generally, but only with myomata; and there is no disquisition on the subject. It is simply a plain unvarnished record of all cases (106 in number) of uterine fibroid treated by the authors according to Apostoli's method up to the date of publication.

This is the first really serious attempt to place before English readers a number of cases treated by thoroughly competent men by means of electricity, and sufficiently large to draw conclusions from. As to the cases themselves, they are evidently recorded with scrupulous honesty; good, bad, and indifferent results being related with equal impartiality; and, in many instances, the patient herself or her medical man being made the mouthpiece. Of the truthfulness of the records there is no doubt. What do we think of the results?

There is but one word to express our feeling—disappointment. We can scarcely ignore the conclusion that, even at the hands of the most skilful men we possess, Apostoli's treatment is tedious, uncertain, and inconclusive; likely to be seen at its best only among the rich and idle of our patients, and not without some risk. That it is tedious, almost beyond tolerance in many cases, there is no gainsaying. That it is uncertain, these records fully prove. And that it is inconclusive is partly shown by recrudescence of symptoms in several cases, and a probable or certain continuance of the tumour in most of those greatly improved.

In all humility, speaking particularly of one who holds deservedly such a pre-eminent position as a man and a surgeon, we cannot admit that, in respect of results attained by electricity, he has made out a certain case of "Surgical hands off." With others, we believe that the full capabilities of the electrical treatment might with safety have been left in non-surgical hands, and that there will always remain cases for which surgical operation will be required. For such cases we want skilled surgeons; and it is a matter of regret to many of us that the capacities of such a man as Keith, unequalled in this domain, should be lost to us. Admitting, as we all do, that the great majority of cases require no treatment; believing, as we must, that some cases are benefitted or even cured by Apostoli's treatment, we maintain that there still remains a small minority which, if surgical interference is ever justifiable as a means of removing suffering, to say nothing of saving life, must be dealt with by surgical removal. And this view is strengthened by a careful study of the work before us.

